# Does nonviolent communication education improve empathy in French medical students?

**DOI:** 10.5116/ijme.615e.c507

**Published:** 2021-10-29

**Authors:** Justine Epinat-Duclos, Alexandre Foncelle, François Quesque, Eric Chabanat, Alexandre Duguet, Jean-Baptiste Van der Henst, Yves Rossetti

**Affiliations:** 1 TRAJECTOIRES Team, Lyon Neuroscience Research Center, INSERM, U1028, CNRS, UMR5292, University of Lyon, France; 2 AP-HP-Sorbonne University INSERM, MRSU 1158, Faculty of Medicine Sorbonne University, Paris, France

**Keywords:** Medical students, empathy, NonViolent communication, perspective taking, social cognition

## Abstract

**Objectives:**

To evaluate the impact of nonviolent communication (NVC) training on five aspects of medical students' empathy skills using implicit and explicit measures.

**Methods:**

312 third-year French medical students were randomly allocated to an intervention group (n = 123) or a control group (n = 189). The intervention group received 2.5 days of NVC training. For each group, empathy-related skills were measured implicitly using three cognitive tests (Visuo-Spatial Perspective Taking, Privileged Knowledge, Empathy for Pain evaluation) and explicitly using two self-rating questionnaires (Jefferson Scale of Physician Empathy, Empathy Quotient). Both groups completed tests and questionnaires before (pre-test) and three months after training (post-test). Responses were collected via online software, and data were analyzed using paired linear mixed models and Bayes Factors.

**Results:**

We found a significant increase in the Jefferson  Scale of Physician Empathy (JSPE) score between pre- and post-tests in the intervention group compared to the control group (linear mixed models: 0.95 points [0.17, 1.73], t_(158) _= 2.39, p < 0.05), and an expected gender effect whereby females had higher JSPE scores (1.57 points [0.72, 2.42], t_(262)_ = -3.62, p < 0.001). There was no interaction between these two factors.

**Conclusions:**

Our results show that brief training in nonviolent communication improves subjective empathy three months after training. These results are promising for the long-term effectiveness of NVC training on medical students' empathy and call for the introduction of NVC training in medical school. Further studies should investigate whether longer training will produce larger and longer-lasting benefits.

## Introduction

It is widely acknowledged that empathy is a powerful tool in the patient-care-taker relationship. When psychotherapists from many different orientations describe their conception of the ideal therapist, they largely agree that empathy is a quality that surpasses all others.^1^^,^^2^ Therapists recognize that the most important factor in being a therapist is to sensitively understand patients from their own point of view.^1^ In this context, empathy has been defined mainly by clinical psychologists and psychiatrists,^2^^-^^4^ who emphasize the distinction between empathy and sympathy, in line with Theodor Lipps’ thinking.^5^ According to Rogers,^2^ “The state of empathy, or being empathic, is to perceive the internal frame of reference of another with accuracy and with the emotional components and meanings which pertain thereto as if one were the person, but without ever losing the "as if" condition.” Although numerous definitions of empathy can be found,^6^ in the clinical context, Rogers proposal carries authoritative weight. According to his definition, empathy includes interest for the other person, the ability to consider their physical, emotional and cognitive states in the here and now, and the capacity to remain one’s self (i.e. to distinguish between self-generated and other-generated percepts). Given this, several basic social cognition skills jointly contribute to processing the multisensory flow of available information that arises from social interactions: visuospatial perspective taking, emotion recognition, knowledge ascription, etc. It is legitimate to think that these social skills are essential in establishing a relationship of trust between caregivers and patients.^2^^,^^7^

While one may expect that practical medical training increases empathic skills, empirical investigations challenge this expectation. Over the last decade, the evolution of medical students’ empathy during the course of their training has been a subject of debate.^8^^,^^9^ In a study by Chen^10^ medical students’ empathy scores evaluated using the student version of the Jefferson Scale of Physician Empathy (JSPE-S) increased by 3 JSPE-S points over the first two years of the preclinical course but declined by 1.75 points between the second and third years of their medical training (i.e. at the start of their clinical experience). Overall, however, no difference was observed between incoming students (113.0) and fourth-year students (113.3), and the decrease remained minor in first-year students within the top tertile of empathy scores. Other studies did not observe a similar decline, with^11^ or without^12^^,^^13^ specific empathy-related interventions, revealing that this effect is not as reliable as is often thought and giving rise to an active debate in the literature. More recently, it was suggested that factors other than the training year play a more influential role. A review of 30 studies^14^ reported a significant trend towards lower empathy scores among medical students across their medical training years, although the most reliable effect that emerged was that female students have higher empathy levels than male students. Additionally, several studies reported a higher level of empathy among students who preferred a “people-orientated” (e.g., psychiatry, general internal medicine, pediatrics) compared to those who preferred "technology-oriented" specialties (e.g., surgery, ophthalmology, anesthesiology, orthopedics and radiology).^15^^- ^^17^ Altogether it seems that the debate over the evolution of empathy throughout the course of medical training should be overtaken by more influential parameters.

Beyond this ongoing issue, there is a growing awareness and consensus of the need to develop empathy training for care-takers and medical students. This is important in order to improve the patient-caregiver relationship as well as to achieve better adherence to treatment and better health outcomes.^7^^,^^18^ A variety of interventions have taken this need into account: visiting patients at home,^19^^,^^20^ writing about the patient’s point of view,^21^ student inpatient hospitalization,^22^ mindfulness skills training,^23^^,^^24^ communication skills training,^25^^-^^34^ simulated experiential learning,^35^^,^^36^ perspective taking training,^37^^,^^38^ theater improvisation teaching,^39^ role playing,^40^^,^^41^ and Balìnt group training.^42^^,^^43^ In the following paragraph, we shall see that several intervention types are likely to increase empathy skills among medical students.

A meta-analysis^44^ of 13 studies showed that empathy may be positively modulated by a range of intervention strategies, among which communication skill workshops showed the greatest quantitative impact on medical students. A subsequent meta-analysis^45^ of 18 studies concluded that several types of interventions (e.g., narrative and creative arts, writing, drama, communication skills training, interprofessional skills training, patient interviews, experiential learning and empathy-focused training) produce positive results, but the authors called for further randomized-control and longer follow-up studies. Confirming these first studies, a recent meta-analysis of 52 studies^46^ identified key elements to improve patients’ perception of the physician empathy: sitting (versus standing) during the interview; detecting patients' non-verbal emotional cues; recognizing and responding to opportunities for compassion; non-verbal communication of caring (e.g., eye contact); and verbal statements of acknowledgement, validation, and support.

These elements underline the critical role of interaction skills, which are combined in a unique manner in the NonViolent Communication processes.^3^ Based on these observations, one may ask whether this approach could improve empathy in medical students.

Inspired by Buddhism's communication ethics^47^ and by his mentor Carl Rogers’^2^ emphasis on empathy in communication and attention to patients’ feelings, the psychologist Marshall Rosenberg^48^ formalized a method for developing the quality of human interactions referred to as Nonviolent Communication (NVC). The NVC model is centered on empathetic listening and honest expressing, i.e., about learning to attend to one’s own deep needs and those of others, as a way to develop compassion. The primary intention of NVC is to foster an empathetic connection between partners.

The practice of NVC is based on four steps. First, observation emphasizes the distinction between observed facts and our more-or-less implicit interpretations, judgments and evaluations, which we are invited to take responsibility for and not let them contaminate the relationship. For example, I do not observe that this little boy is nasty, as I can only observe that he ate the cake that was in another child’s hand; therefore, it is only my interpretation that “he is nasty”. Second, NVC invites us to attend deeply to our present feelings, which may be sensed throughout our bodies. For example, I may feel sad, shocked, angry, amused, etc, by this scene. The third step is to take responsibility for our feelings by connecting them to our deep needs. For example, I may feel sad because I imagine the other child is hungry, and a need for equity is acknowledged in me right now, or I may feel angry because a need for justice is alive in me, or I may feel amused because I connect the child’s innocence to my need for lightness. The interesting idea about needs is that we should ensure that they reflect universally shared needs (e.g., a need for water, movement, intimacy, support, serenity or expression). Fourth, the unique part of NVC is to prolong this process by expressing a request. This request should be specific and fundamentally target the connection between the people involved. It may, for example, include asking for a reformulation, expressing interest in the other’s reactions, feelings and needs, etc. This connection is crucial, as it opens doors to possible actions that individuals may undertake jointly (or not). The other part of the communication process consists of receiving similar pieces of information from the other person: observation, feelings, needs and requests.

About 20 years ago, Rosenberg articulated the promising effects of NVC in medical doctors and care-takers.^3^ However, there is no clear empirical evidence for the positive effect of NVC on medical doctors' empathy. It is important to note that although NVC has become increasingly popular, very few experimental studies have investigated its effects. Among these, we shall distinguish between those using a qualitative methodology (e.g., subjective comments)^49^^,^^50^ and those using a quantitative methodology (e.g., questionnaires)^51^^-^^55^ as well as between those conducted in a medical or non-medical context.

Regarding qualitative observations of NVC education on students in an e-mentoring context and using a case study approach, Cox and Dannahy^49^ described that the NVC process improved trust in the personal relationships between a small group of three university students and their tutor and was characterized by greater openness. In another case, based on stories from nursing student diaries, Nosek^50^ reported that NVC reinforces the ideal of authenticity through dialogue. Another study examined the impact of NVC training in the Hispanic community of Northern California, but the low number of respondents (i.e., n=13) prevented them from conclusive whether NVC training improved empathy and self-efficacy in conflict management.^54^

Regarding the influence of NVC in the medical context, three studies are relevant to the current work. First, a mixed-methods study showed a positive effect of a brief NVC training (two 105 minute sessions) on the empathy of 55 nursing students (measured using the Interpersonal Reactivity Index).^56^ Qualitative analyses suggested an increase in empathy towards oneself and others after the training. Museux and colleagues^53^ explored the effects of a single 7-hour NVC training on the interprofessional collaboration of 9 care-takers. They reported an improvement in group skills in terms of decision-making and sharing action plans, even if there was no significant improvement in communication skills. More recently, Wacker and Dziobek^55^ investigated the effect of NVC on interpersonal skills among health professionals (46 trained people and 43 controls). They reported greater verbalization of negative emotions during a conflictual group discussion following a 3-day NVC training.

Furthermore, improved NVC skills in everyday communication remained after three months.  A recent study used a 500-contact-hour curriculum integrated over four years, consisting of training in mindfulness, cognitive behavioral therapy, nonviolent communication, motivational interviewing, spirituality in healthcare, wellness, equanimity, and 'being with suffering'.^57^ A total of 258 medical students volunteered and received serial self-assessments. They reported continued growth in personal development, professional development, and the “ability to empathize and connect with others”.^57^ Altogether several qualitative and quantitative arguments point to a positive effect of NVC on interpersonal relationships or representations.

The above literature review reveals that NVC has a clear potential to increase empathy among care-takers as it targets the key parameters that contribute to perceived empathy in patients.^46^ However, there is no empirical evidence showing that NVC improves empathy in medical students. In the present study, we investigated the effects on selected social interaction skills of a brief NVC training program administered to medical students. Studies of medical student empathy are hampered by various definitions of empathy, limited sample sizes, lack of adequate control groups, and the use of a variety of empathy measurement instruments.^44^^,^^45^^,^^18^ In an attempt to overcome these difficulties and to identify which aspects may be affected by NVC training, we measured several aspects of empathy-related skills using a variety of explicit (self-assessment questionnaires: Jefferson Empathy Scale, Empathy Quotient) and implicit (efficiency tests: spatial perspective-taking, empathy for pain, privileged knowledge) measures.^58^^-^^60^ Furthermore, we designed our study to include a large cohort of students, randomly assigned to an intervention or control group, as in clinical trials.^61^^,^^62^

## Methods

### Study design, participants and setting

**Figure 1:**
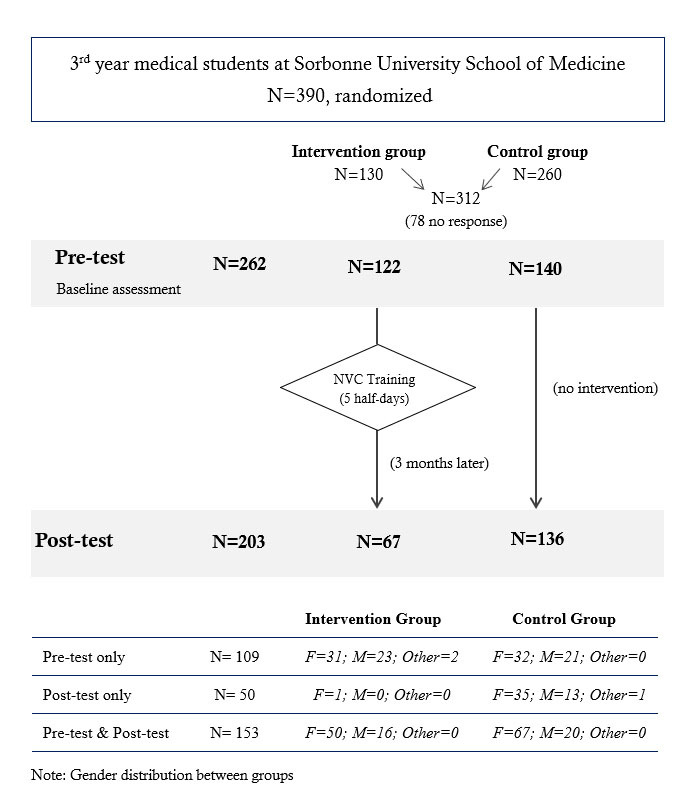
Study design and participants

We performed this experimental study with medical students during the academic year 2018-2019. Students were recruited from the 390 third-year medical students enrolled at the Faculty of Medicine of Sorbonne University (Paris). Out of these 390 students, 312 volunteers (median age: 22 years) were randomly allocated to attend an intervention group (n=123) or a control group (n=189). The intervention group received five half-days of NVC training, and the control group had no NVC intervention but instead received training in cardiology and neurology (see Figure 1). Testing was carried out both before (pre-test) and three months after (post-test) the NVC training. A total of 153 of the initial 312 students participated in both the pre-and post-testing sessions. The remaining students participated in either the pre-or post-test. Compared with the literature, our sample size of 312 medical students is in the higher range of available controlled studies of medical students (13 to 299)^18^ or studies of the effect of NVC (3 to 89 (see introduction above)). This study was approved by the Sorbonne medical school, and participants were informed that this questionnaire and the use of its results were anonymous. According to the Declaration of Helsinki, participants gave their consent to participate in this anonymous online study.

Figure 1. Study design and participants

### Intervention: Content and course design

NVC training was delivered by a trainer certified by the Center for Nonviolent Communication. Introductory information and general principles were delivered to the whole group in the amphitheater, and most of the practice was done in sub-groups of 12 students. The aim of the training was to explain the NVC model and principles, give experience and practice in self-awareness for feelings and needs, and provide opportunities to explore personal motivations and intentions as well as being receptive to the other’s feelings and needs. The four steps of the NVC process (Observation, Feelings Needs and Requests) were explained and practiced, and several basic interaction modes were explored. Participants gradually learned to be more aware of their own feelings and needs and eventually understood that all people have the same universal needs. Practice was aimed at distinguishing between observation and interpretation, between need and strategy, between feeling and masked evaluations, and between requests and demands. During the various training sessions, participants practiced alone or were invited to exchanges in pairs or small groups. Different practical exercises were proposed as mirror reformulations (i.e. "You say...") and listening with three possible attitudes (silence, simple reformulating, reformulating with focus on the speaker's feelings and needs).

### Jefferson Scale of Physician Empathy (JSPE)

The JSPE was designed to measure the self-reported level of empathy of health professionals in a clinical setting.^63^ Here, we used a French version of the JSPE^64^ but retained only the five items that are relevant to medical students and common to the physician and student versions of the JSPE. Scores were calculated with a seven-point Likert scale ranging from "Strongly disagree" to "Strongly agree".

**Table 1:** Student ratings for pre - and post-test for the intervention group

Learning gain	Intervention Group				t or z	p-value
	Pre		Post			
	M	SE	M	SE		
Self-administered questionnaires						
Jefferson Scale of Physician Empathy (JSPE)	31.14	0.33	32.09	0.43	2.39	0.02*
Empathy Quotient (EQ)						
Cognitive Empathy (CE)	10.45	0.33	10.23	0.41	-0.63	0.53
Emotional Reactivity (ER)	10.54	0.32	9.88	0.39	-1.96	0.05
Cognitive task						
Visuo-Spatial Perspective Taking (VSPT)						
Right	-0.86	0.68	-1.54	0.84	-0.96	0.34
Left	-24.11	4.44	-11.05	2.10	3.87	0,0001*
Both	-10.31	1.82	-13.01	4.38	-0.71	0.48
Privileged Knowledge (PK)						
Medical stories	4.04	0.16	4.21	0.22	0.75	0.46
Daiy stories	3.30	0.19	3.00	0.27	-1.02	0.31
Pain intensity						
Pen pictures	0.93	0.12	0.92	0.16	-0.04	0.97
Needle pictures	2.87	0.19	2.92	0.26	0.21	0.84

**Table 2:** Student ratings for pre- and post-tests for the control group

Learning gain	Control Group				t or z	p- value
	Pre		Post			
	M	SE	M	SE		
Self-administered questionnaires						
Jefferson Scale of Physician Empathy (JSPE)	30.86	0.33	30.73	0.33	-0.39	0.70
Empathy Quotient (EQ)						
Cognitive Empathy (CE)	9.88	0.33	9.66	0.33	-0.71	0.48
Emotional Reactivity (ER)	10.68	0.31	10.22	0.31	-1.62	0.11
Cognitive task						
Visuo-Spatial Perspective Taking (VSPT)						
Right	-2.11	1.05	-1.40	0.79	1.02	0.31
Left	-23.06	4.49	-10.99	1.70	3.39	0,0007^*^
Both	-9.76	1.71	-12.28	2.89	-0.98	0.33
Privileged Knowledge (PK)						
Medical stories	3.99	0.16	3.92	0.16	-0.37	0.71
Daiy stories	3.45	0.20	3.40	0.20	-0.21	0.83
Pain intensity						
Pen pictures	1.15	0.12	1.06	0.12	-0.62	0.53
Needle pictures	2.90	0.19	2.90	0.20	0.01	0.99

### Empathy Quotient (EQ)

The EQ is a self-assessment test that measures empathy in adults.^65^ As a former factorial analysis identified three sub-sections in the EQ; social skills, cognitive empathy, and emotional responsiveness.^66^ We selected the top ten most informative questions of the cognitive empathy and the emotional responsiveness sub-sections, i.e., our questionnaire included 20 items. For each item, participants were requested to rate to what extent they agreed or disagreed on a 4 points scale ranging from "Strongly disagree" to "Strongly agree".

### Visuospatial Perspective Taking (VSPT)

VSPT was investigated with a simple test that consisted of asking participants to describe where an object is located with respect to another object in a visual scene. Specifically, the participants were presented with a picture displaying a character sitting at a table with a water bottle and a book, and they were required to describe the relationship between the two objects on the table. Depending on the spatial descriptions obtained, it was possible to determine if participants used their own perspective (e.g., “at my left”) or spontaneously endorsed the perspective of the other person (e.g., “at his/her right”), or mentioned both (e.g., “at its right from the character’s point of view or at its left from mine”). It is now considered that this test measures the propensity to spontaneously consider the visuospatial perspective of another person.^67^^-^^71^

### Privileged Knowledge (PK)

The ability to infer others’ mental states was investigated using the Privileged Knowledge paradigm.^58^^,^^68^^,^^72^ Communication is often ambiguous, and to resolve ambiguity, the speaker and the addressee need to consider what the other knows and does not know. Experimental evidence shows that people who have privileged information about speakers’ intentions tend to assume that other listeners will make the same interpretation as them despite the fact that the other listeners do not have access to this privileged information.^72^ They thus overestimate the transparency of speakers’ utterances and inaccurately predict that others will interpret the content of their speech in the same way as they do. We designed this test with two types of stories evoking either a daily life context or a medical context. In the daily life stories, short interactions were described (e.g., X thanks Y for a restaurant that he recommended), and participants had to estimate to what degree the speaker is perceived as ironic by the receiver (as in Keysar, 1994)^72^ while having themselves access to privileged information (e.g., the speaker had an awful experience). In the medical stories, a relative's symptoms are described to the family physician (e.g., X, who worries about Y and calls the doctor to report Y’s symptoms). Participants had to estimate to what degree the doctor would evaluate the patient’s state as severe while having themselves access to privileged information (e.g., a third symptom, not reported during the phone call, but very typical of a serious illness). For each type of story, we designed four versions. Scores were calculated using a seven-point Likert scale from "Benign problem" to "Serious problem" for the medical stories and from "Not at all interpreted as ironic" to "Totally interpreted as ironic" for daily life stories. Participants were randomly assigned to a different version of each type of story for each assessment.

### Empathy for Pain evaluation

Our test of empathy for pain was based on the work of Xu and colleagues^73^ and used pictures of males who had either a syringe needle or a ballpoint pen tip applied to their right cheek. Participants were asked to rate the pain felt by the individual in the picture. Images were presented randomly and displayed males of two different ages (30 vs 55) and two different ethnic origins (European vs Asian). Similar to the analogical scale for pain,^74^^-^^76^ scores are calculated using a nine-point Likert scale from "Not painful at all" to "Extremely painful".

### Data collection

Responses were collected over one month for both testing sessions, from 10 September 2018 to 8 October 2018 for the pre-interventional survey (pre-test) and from 21 January 2019 to 25 February 2019 for the post-interventional survey (post-test). NVC training was administered over five consecutive mornings from 17 to 21 September 2018. Even if the pre-test period and the training period marginally overlapped, we ensured that the training participants had all performed the pre-test before their training.  In order to obtain more robustness in our results, the order of the test versions was counterbalanced across participants.

### Data analysis

The data were pre-treated, removing missing values (9%), reorganizing data in clear data frames and calculating scores for Jefferson scale and Empathy Quotient with Python3 programming language.^77^ All analyses and figures were processed with R-programming language.^78^ We constructed linear mixed models and generalized linear binomial models and tested mean differences with posthoc pairwise comparisons between control and test groups. This type of model takes into account the repetition of several tests for the participants in one session and the repetition of sessions. In addition, we validated each model by verifying the normality of the residuals and calculating a conditional R^2^ designed for mixed models. Finally, in order to make a model selection, we used a Bayesian approach with the version 2.14.4 of the 'brms' (Bayesian Regression Models using 'Stan' Version) package for R.^79^ Indeed, we computed Bayes Factors, meaning the likelihood ratio of two competing models, in order to select the model for hypothesis testing.^71^

## Results

Out of the 390 third-year medical students, 262 (67.2%) participated in the first testing session, and 202 (51.8%) in the second test (χ^2^_(1)_ = 8.23, p < 0.01). A total of 153 students successfully completed both questionnaires: 66 (50 females) of them followed the NVC training between pre-and post-test, whereas 87 (67 females) did not.

### Questionnaires

#### Jefferson Scale of Physician Empathy 

**Figure 2:**
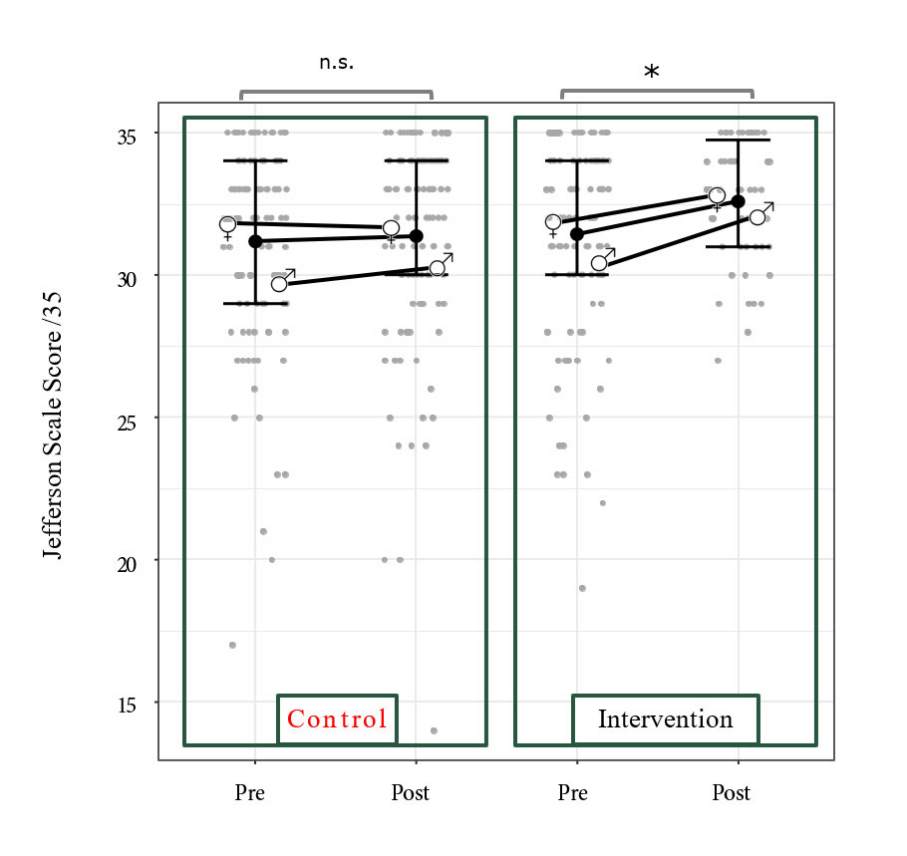
Distribution of the JSPE subscore

**Figure 3:**
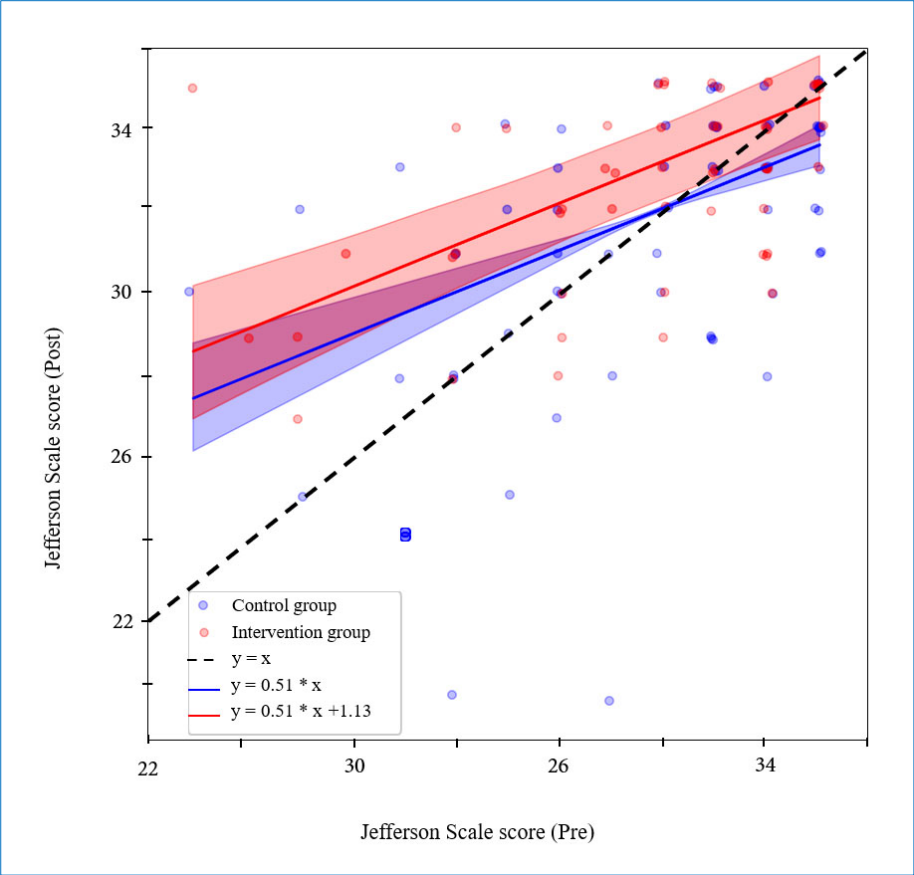
Correlation of JSPE scores between pre and post sessions

In the control group (275 valid responses), scores at pre-test remained stable at post-test (around 32). In the NVC group, our JSPE subscore increased significantly from 32 to 35 between the pre-and post-tests. Figure 2 shows data from each group with different symbols for male and female participants. The difference between mean pre-and post-test scores was significant in the intervention condition only. Statistical comparisons by gender are provided in Table 4. We used multivariate linear mixed models with paired analysis to compare the JSPE scores pre-and post-training in the control and NVC-trained participants. As shown in Figure 2, skewed distributions were observed with a ceiling effect that might have prevented us from obtaining a significant improvement. The interaction variable between the group and the session was significant (see Figure 2 and Table 3). JPSE score increased by 0.95 points (95% confidence interval (CI) [0.17, 1.73], t_(158)_ = 2.39, p < 0.05) in the JSPE score between pre-and post-tests in the intervention group while the control group showed no significant increase between the two test sessions (-0.13 points, t_(174)_ = -0.39, p = 0.7 see Table 1 and Table 2). In addition, group and session factors alone were not significant effect (Group: 0.28 points with a 95% CI [-0.59, 1.14], t_(342) _= 0.62, p = 0.53 and Session: -0.13 points with a 95% CI [-0.78, 0.53], t_(154)_ = -0.392, p = 0.70, respectively). This model had a conditional R^2^ equal to 0.61. Next, we conducted the Bayes Factor analysis by comparing models with and without the interaction term between Group and Session variables. It turned out that only the Bayes factor for the Jefferson scale showed a ratio, namely 2.83, in favor of the model with the interaction term. We also used the same model to analyse each item of the JSPE score separately, with no significant outcome. In addition, there was a significant effect of gender; on average meaning females exhibited higher scores than males (1.57 points with a 95% CI [0.72, 2.42], t_(262)_ = -3.62 , p < 0.001). Last, there was no significant interaction between gender and Group or Sessions. A final supplementary analysis ensured that the students did not memorize their initial pattern of response in order to reproduce it during the second session. Figure 3 illustrates the result of the Bayesian linear regression and associated credible interval. Overlapping points are presented with a jitter for the sake of clarity. Based on the general pattern it can be concluded that participants did not simply tend to memorize and reproduce their responses over the two tests.

#### Empathy Quotient

We also used a linear mixed model with paired analysis to compare the EQ score between pre-, post-test and intervention and control participants. The variables associated with both the Cognitive Empathy (CE) and the Emotional Reactivity (ER) scores, gender, group, the session and their interactions did not yield any significant results. These models had a conditional R^2^ equal to R^2^ = 0.71 (p < 0.001) and R^2^= 0.74 (p<0.001), respectively. In addition, the complementary Bayes Factor analysis favored the models without a Group and Session interaction (see Table 3).

**Table 3:** Summary statistics for Session and Group variables in the multivariate linear mixed model and Bayes Factor analyses

Interaction variable between group and session	Interaction	Standard error	t or z value	p-value	Bayes Factor
Self-administered questionnaires					
Jefferson Scale of Physician Empathy (JSPE)	1.03	0.38	2.71	0.007^**^	2.83
Empathy Quotient (EQ)					
Cognitive Empathy (CE)	-0.19	0.37	-0.52	0.61	0.41
Emotional Reactivity (ER)	0.12	0.38	0.33	0.74	0.47
Cognitive task					
Visuo-Spatial Perspective Taking (VSPT)					
Right	-0.93	0.60	-1.55	0.12	0.82
Left	-0.20	0.55	-0.37	0.71	0.90
Both	0.61	0.65	0.94	0.35	0.74
Privileged Knowledge (PK)					
Medical stories	0.01	0.33	0.02	0.98	0.33
Dairy stories	-0.18	0.35	-0.53	0.60	0.49
Pain intensity					
Pen pictures	0.11	0.22	0.49	0.63	0.18
Needle pictures	0.13	0.34	0.38	0.70	0.31

##### Cognitive tests

Performance in the Visuospatial perspective taking (VSPT), the Privileged Knowledge and the Empathy for pain tasks did not significantly evolve between the pre-and post-test (see Table 1 and Table 2): the complementary Bayes Factor analyses favored for the models without interaction terms between-group (intervention group and control group) and session (pre-and post-test) (see Table 3).

**Table 4:** Summary statistics for pre- versus post-test JSPE values

Group	Estimate	SE	t value	p-value
Control				
Women	-0.16	0.38	-0.41	0.69
Men	-0.06	0.70	-0.09	0.93
Intervention				
Women	0.82	0.46	1.77	0.08
Men	1.38	0.80	1.71	0.09

## Discussion

This study was designed to evaluate the effects of a brief NVC training on empathy-related skills (linked to the visuospatial, emotional and cognitive domains) in a large group of French medical students. Explicit and implicit levels of processing can be dissociated in both perception^80^ and action^81^^,^^82^ fields, and this also holds for social cognition.^60^^,^^83^ As explicit questionnaires and implicit tests were used, this study enabled us to test whether only explicit or both explicit and implicit evaluations were affected. A plausible hypothesis was that the short-term effects of this brief training might lead to better explicit evaluation (i.e., awareness measures) than implicit evaluation (efficiency tests). Our main result is that the Jefferson Scale subset score increased significantly, whereas the other tests did not show any significant variation. These primary results will be discussed below.

First, our student sample size (N = 312) is much larger than that used in previous NVC assessment studies^51^^,^^53^ and comparable to several studies on empathy in medical students.^84^^,^^85^ The percentage of response was respectively 67.2% and 51.8% for the pre and post-tests, which is moderately successful. This large sample enabled us to draw reliable conclusions about the specific presence of the effect found on the Jefferson Scale in spite of skewed distributions combined with a ceiling effect. 

Overall, only the JSPE score exhibited a significant increase between the two sessions in the intervention group. Interestingly, every individual item from our sub-score exhibited a similar profile to the between-group result across time, which calls for the reliability of the effect reported here. The subset of the Jefferson Scale exhibited a significant increase (by about one point) in the intervention group, whereas it did not significantly evolve (+ 0.18) in the control group. Considering the distribution of this variable (Figure 2), such a positive outcome may appear unlikely as it shows a clear ceiling effect, which could have prevented us from detecting a significant effect. Additionally, such a ceiling effect is not apparent for the other variables.

Therefore, the significant result obtained here appears to be robust, and the effect size reinforces this idea. In addition, Bayes factors for all tests except JSPE were inferior to 1 (see Table 3), meaning that models without a term for the interaction between the group and the Session variables are more appropriate. Indeed, the Bayes factors indicated "moderate evidence" in favor of the model with the interaction term against the model without the interaction term if their value is close or above 3, or the reverse if it is close or below 1/3. This threshold between anecdotal evidence and moderate evidence for one model in favor of another model is used to draw a practical parallel with p-value thresholds.^86^Thus, as the Bayes factors for the JSPE models was almost 3 (in favor of the interaction model). As Bayes factors for all other tests are inferior to 1, it is safe to conclude that the JSPE changed significantly due to NVC training and that this training has no effect on the other tests used here. In addition, the tests with Bayes factors below ⅓ show "moderate evidence" in favor of the model without the interaction term, meaning that the corresponding results are significantly negative.

As our subset of the JSPE (5 items) was custom adapted for the sake of test duration, it is not possible to directly compare the magnitude of our results (one-point gain for five items) with previous studies. However, the increase in this score appears to be in line with previous studies. Our 1 point gain for five items matches the 4 points gain out of 20 items (4/20) which were observed in 488 French medical students trained via forum theatre and tested after the training sessions.^39^ Using another communication skill training in India, D'Souza showed that a single training session transiently but significantly improved the JSPE score by 4.5/20.^34^

One of the main concerns for both adaptation^87^ and readaptation^75^ programs is the duration of the effects. It is also important to emphasize that our post-training test was administered more than 3 months after the NVC training, which suggests that sustained effects may be obtained following short exposure. In the available literature, most studies reporting the effect of interventions aimed at improving medical students' empathy did not explore the duration of the benefit. Two studies mentioned no differences on the JSPE, 7 or 26 days after drama intervention,^41^ 3 months^88^ and 12 months following Balint group training.^42^ Only a few studies reported an increase in JSPE 1 week following seven sessions of 1.5-hour Balint groups distributed over three months^43^ and five weeks following a 2-hour empathy workshop^89^ or even a decrease three weeks after 2-hour empathetic communication skills.^34^  Following the brief intervention used in our study, it is interesting to observe that the durability of the effect is surprisingly long. Therefore, our NVC training procedure appears to yield promising results in terms of effect duration following short grouped training. Congruently, Wacker and Dziobek^55^ also reported a three-month effect on self-evaluation questionnaires in a non-randomized sample of 29 public health employees (physicians, nurses and administrative personnel) who volunteered for NVC training.

The duration of the effect of such short interventions may be prolonged with more substantial training. In this vein, Kramer and colleagues^90^ observed an increase in supportive behaviors among groups of students only and students with tutors up to 6 and 12 months following a five week distributed training. It thus remains to be investigated whether distributed NVC training further increases the retention delay for the beneficial effect on empathy and produces durable effects.

### Limitations

One limitation of our study is that we only observed an effect of the NVC intervention on the subjective measures. Therefore it remains to be investigated whether longer training in NVC may affect not only explicit measures but also more implicit tests. In addition, the duration of the follow-up was arbitrarily chosen here for exploratory purposes. Moreover, the fact that significant effects are observed at three months post-intervention calls for investigating several time points before and beyond this interval in order to characterize the time course of the benefit obtained and determine whether the acquired benefit is likely to grow or decline over time.

The current study provides a global evaluation of classic NVC training. When reading the literature, it is apparent that particular types of interventions on medical students have been investigated by a large number of studies: perspective taking,^91^^,^^92^ patient's^93^^,^^94^ and one’s own^95^^,^^96^ emotions and feelings recognition,^97^^,^^98^ identifying the patients' needs,^99^^,^^100^ avoiding judgments,^24^^,^^101^ role-playings.^30^^,^^102^ It is interesting to note that these interventions tend to develop the many facets of empathy, as defined by Carl Rogers (1995): « entering the private perception world of the other, avoiding judgment, checking the accuracy of your perception by putting aside opinions and values you have for yourself in order to enter the world of others without prejudice, being sensitive to the feelings and meanings, accurately perceiving the emotions of others, identifying the needs of others ».^2^ The specificity of the NVC approach is that it offers a simple operationalisation of a process that aims to increase sensitivity towards oneself and others. Once positive effects on empathy are confirmed, it will be interesting to determine which component of the NVC training (which includes not only four steps but also important distinctions between observations and interpretations and between query and demands) crucially contributes to the benefit observed here. Such understanding will determine which components should be included or even developed in future empathy training courses.

In our study, neither the Cognitive Empathy (CE) component nor the Emotional Reactivity (ER) component of the Empathy Quotient (EQ) score was improved following the NVC intervention. A possible explanation for this is that the JSPE focuses explicitly on the relationship with patients, which was highlighted in the NVC training of the students, whereas the EQ was developed to detect pathological deviations. Questions from the CE and ER assess relationships in general and across the participant's whole life and are therefore less likely to be altered in the short term. Interestingly our measures of visuospatial perspective-taking and mental state inference also did not exhibit significant changes before and after the NVC training. This raises the question of whether prolonged training in NVC may also improve these abilities or whether NVC training specifically influences social interaction abilities linked to the emotional domain. 

## Conclusions

Taken altogether, the specific improvement of the score on the Jefferson Scale of Physician Empathy suggests that the brief training delivered to the students enabled them to gain awareness about the role and importance of empathy in their relationship with patients. It is reassuring and promising that this effect lasted for at least three months. A further challenge will be to evaluate whether deeper training through a longer and distributed course may expand the present results in the form of amelioration of efficiency tests as well.^58^

Data from the present study encourage the introduction of NVC training in medical schools and motivate future studies. Knowledge about its positive effects and implications for an improved therapeutic alliance should increase medical students’ interest and personal involvement in this training.

### Acknowledgements

The authors wish to thank Karen Reilly for copyediting the manuscript, Sonia Alouche, Jean-Louis Borach, Anne Cheylus, Eric Koun, Romeo Salemme and Frédéric Volland for their valuable technical assistance and Véronique Gaspard for the NVC training coordination. This work was supported by Inserm, CNRS, HCL, Lyon University and the Faculty of Medicine Sorbonne University. 

### Conflicts of Interest

The authors declare that they have no conflict of interest.

## Supplementary material

Supplementary file 1 Appendix. Items rated in the online survey
