# Supplementary material for: Does nonviolent communication education improve empathy in French medical students?
Source: Int J Med Educ. 2021 Oct 29;12:205–18. doi: 10.5116/ijme.615e.c507 (PMC8994647; doi:10.5116/ijme.615e.c507)
Supplement: Supplementary file 1 — Appendix. Items rated in the online survey [file ijme-12-205-S1.pdf]

Appendix A

Items rated in the online survey

Self-administered questionnaires			Achievable points
Jefferson Scale of Physician Empathy (JSPE) (Hojat et al. 2002)			35
1	I try not to pay attention to my patients' emotions in interviewing and history taking	Likert scale (1-7)	
2	Patients' illnesses can be cured only by medical or surgical treatment; therefore, a physician's emotional involvement does not have a significant role in treatment	Likert scale (1-7)	
3	Asking patients about what is happening in their personal lives is not helpful in understanding their physical complaints	Likert scale (1-7)	
4	Emotion has no place in the treatment of medical illness	Likert scale (1-7)	
5	I do not enjoy reading non-medical literature and have no involvement with the arts	Likert scale (1-7)	
Empathy Quotient (EQ) (Baron-Cohen, 2004)			40
Cognitive Empathy (CE) sub-parts			20
1	I can tune into how someone else feels rapidly and intuitively.	2	
2	Other people often say that I am insensitive, though I don't always see why.	2	
3	I am good at predicting how someone will feel.	2	
4	Seeing people cry doesn't really upset me.	2	
5	I am good at predicting what someone will do.	2	
6	If I say something that someone else is offended by, I think that that's their problem, not mine.	2	
7	I can tell if someone is masking their true emotion.	2	
8	I am able to make decisions without being influenced by people's feelings.	2	
9	I can easily work out what another person might want to talk about.	2	
10	I really enjoy caring for other people.	2	
Emotional Reactivity (ER) sub-parts			20
11	Other people tell me I am good at understanding how they are feeling and what they are thinking.	2	
12	I am very blunt, which some people take to be rudeness, even though this is unintentional.	2	
13	I can pick up quickly if someone says one thing but means another.	2	
14	I get upset if I see people suffering on news programs.	2	
15	I can easily tell if someone else is interested or bored with what I am saying.	2	
16	People sometimes tell me that I have gone too far with teasing.	2	

17	I can sense if I am intruding, even if the other person doesn't tell me.	2	
18	I usually stay emotionally detached when watching a film.	2	
19	Friends usually talk to me about their problems as they say that I am very understanding.	2	
20	I tend to get emotionally involved with a friend's problems.	2	
Cognitive tests			
Visuo-Spatial Perspective Taking (VSPT) (Quesque et al. 2018) "with respect to the bottle, where is the book?"		3 types of response Right, Left, Both	
Privileged Knowledge (PK) (Keysar 1994; Todd et al. 2011)			16
Medical stories (4 versions, see 1 example below)		Likert scale (1-7)	
"Mr. Luquet, 67, has been experiencing headaches, dizziness and falls for several weeks. He refuses to consult. His daughter, who sees him regularly, decided to talk to the family doctor. She writes him a text message: "My father has been having headaches for a few weeks. I feel worried about it."			
Dairy stories (4 versions, see 1 example below)		Likert scale (1-7)	
"François wants to test a new bar with his group of friends. Alexis warmly recommends a specific one to him, adding that he won't be disappointed. On D-Day, François and his friends have a terrible time in this bar. The music is too loud to hear each other without much effort and the table is sticky. The next day, François writes a text message to Alexis: "Thanks for the good recommendation! I know I can rely on you in the future!"			
Pain intensity (Xu et al. 2009)			18
(2 items, one with a pen and the other with the needle) "In your opinion, what level of pain do you think this person is experiencing?".		Likert scale (1-9)	
